# Cardiac sarcoma presenting as paraneoplastic arthritis and clubbing: a case report and literature review

**DOI:** 10.1186/s41927-023-00350-x

**Published:** 2023-09-15

**Authors:** Natasha Ung, Carmella Gunasingam, Ken Cai, Nicholas Manolios, Angela Bayly, Phuong Dinh, Yishay Orr, Preeti Choudhary, Peter Wong

**Affiliations:** 1 Department of Rheumatology, Westmead Hospital, Cnr Hawkesbury Road and Darcy Road, Westmead, NSW 2145 Australia; 2 Westmead Clinical School, Faculty of Medicine and Health Sciences, University of Sydney, Sydney, NSW Australia; 3 Department of Tissue Pathology and Diagnostic Oncology, Westmead Hospital, Westmead, NSW Australia; 4 Department of Medical Oncology, Westmead Hospital, Westmead, NSW Australia; 5 Department of Cardiothoracic Surgery, Westmead Hospital, Westmead, NSW Australia; 6 Department of Cardiology, Westmead Hospital, Westmead, NSW Australia; 7 Rural Clinical School, University of New South Wales, Coffs Harbour, NSW Australia

**Keywords:** Cardiac sarcoma, Arthritis, Paraneoplastic syndrome

## Abstract

**Background:**

Cardiac tumours are rare, and clinical manifestations depend on the anatomical location. Symptoms can be the result of cardiac outflow anomalies, constitutional features such as fever, loss of weight, and/or paraneoplastic manifestations such as arthritis. To date, there has only been one other case report in the literature of cardiac sarcoma presenting as paraneoplastic arthropathy.

**Case presentation:**

A 52-year-old woman presented with acute onset corticosteroid-resistant inflammatory polyarthralgia, clubbing and a systolic murmur. Transthoracic echocardiogram revealed a dilated left atrium with an echogenic mass and brain magnetic resonance imaging revealed multiple embolic infarcts. Histopathology following emergency resection showed a Grade 3 left atrial intimal sarcoma. The polyarthralgia and clubbing resolved soon after tumour removal. The patient went on to receive chemotherapy and remains in remission.

**Conclusions:**

This case highlights the rare paraneoplastic association of cardiac sarcoma and arthropathy.

## Background

Primary cardiac tumours are rare, with only one tenth being malignant [1]. Clinical manifestations depend on the anatomical location of the tumour. Symptoms can also arise from embolic, constitutional and paraneoplastic manifestations. If tumours are large enough, they can cause symptomatic outflow obstruction with syncope and shortness of breath. The most frequent cardiac malignant tumours are sarcomas, which are rare, and aggressive [1] - requiring urgent surgical intervention [2]. There has only been one other case of cardiac sarcoma associated with arthritis [3]. We present the second case report of a cardiac sarcoma manifesting as paraneoplastic polyarthralgia.

## Case presentation

A previously well 52-year-old woman was reviewed in our Rheumatology Clinic via telephone consultation due to SARS-Cov-2/Covid-19 restrictions. The main presenting symptom was a 3-month history of severe inflammatory arthralgia involving both hands, wrists, knees and ankles. Symptoms were attributed to the BNT162b2 Covid-19 mRNA vaccine which she had received 1 week prior. In view of this possibility, the ChAdOx1-S Covid-19 vaccine was administered as a subsequent dose, 3 months following the initial BNT162b2 mRNA vaccine. A 5-day course of prednisolone at a dose of 5 mg daily was commenced by her general practitioner just prior to administration of the ChAdOx1-S vaccine to prevent further arthralgia. At review by telephone, 1 month later, the arthralgia had worsened, and she was commenced on prednisolone 15 mg daily for symptomatic relief. There were no constitutional, cardiac or respiratory symptoms.

Investigations performed prior to the telephone consultation revealed an elevated C-reactive protein (CRP) of 101 mg/L (normal < 5 mg/L); erythrocyte sedimentation rate (ESR) of 64 mm/hr (normal < 14 mm/hr). Rheumatoid factor (RF); anti-cyclic citrullinated peptide (anti-CCP), antinuclear antibodies (ANA) and extractable nuclear antigens (ENA) were negative. On review 2 weeks later, again by telephone due to Covid-19 restrictions, there had been little improvement with prednisolone and a face-to-face consultation was arranged for more complete assessment. This was undertaken 2 weeks later by a rheumatologist in full personal protective equipment.

**Fig. 1:**
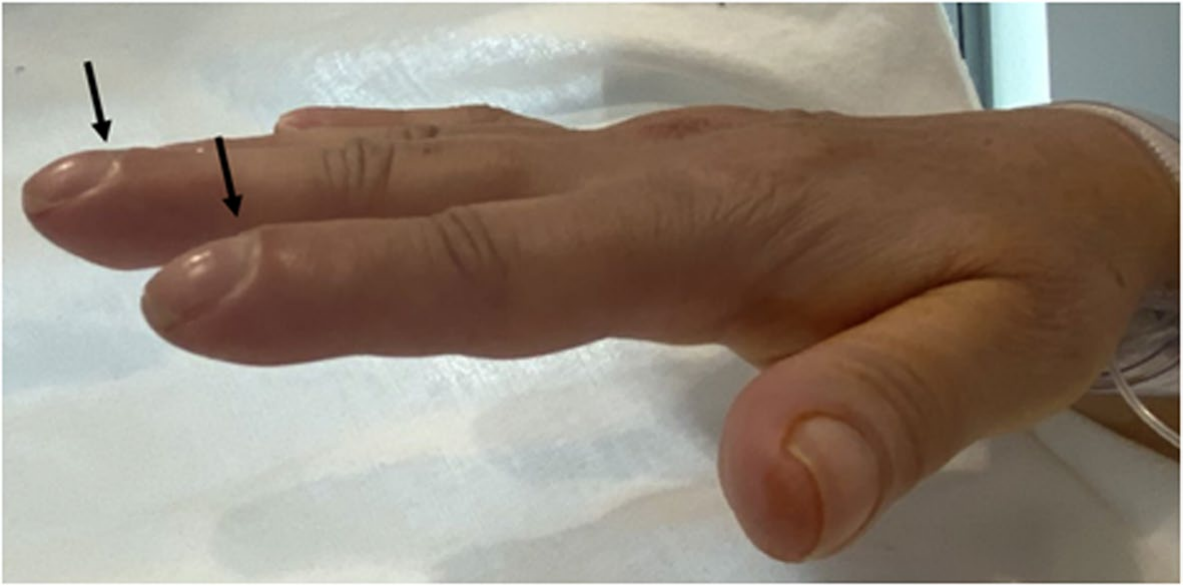
Digital clubbing affecting the right hand (black arrows)

On examination, the patient was mildly dyspnoeic, with a temperature of 38°C and a resting heart rate of 100 beats/min. Examination of the musculoskeletal system showed marked bilateral wrist swelling, non-tender bilateral knee effusions and bilateral swollen ankles. Notable was the presence of marked bilateral fingernail clubbing (Fig. 1), a loud apical systolic murmur, right ventricular heave and bi-basal crepitations. (The patient astutely recalled the clubbing had only commenced when she became unwell 3 months prior). Peripheral stigmata of infective endocarditis were absent.

**Fig. 2:**
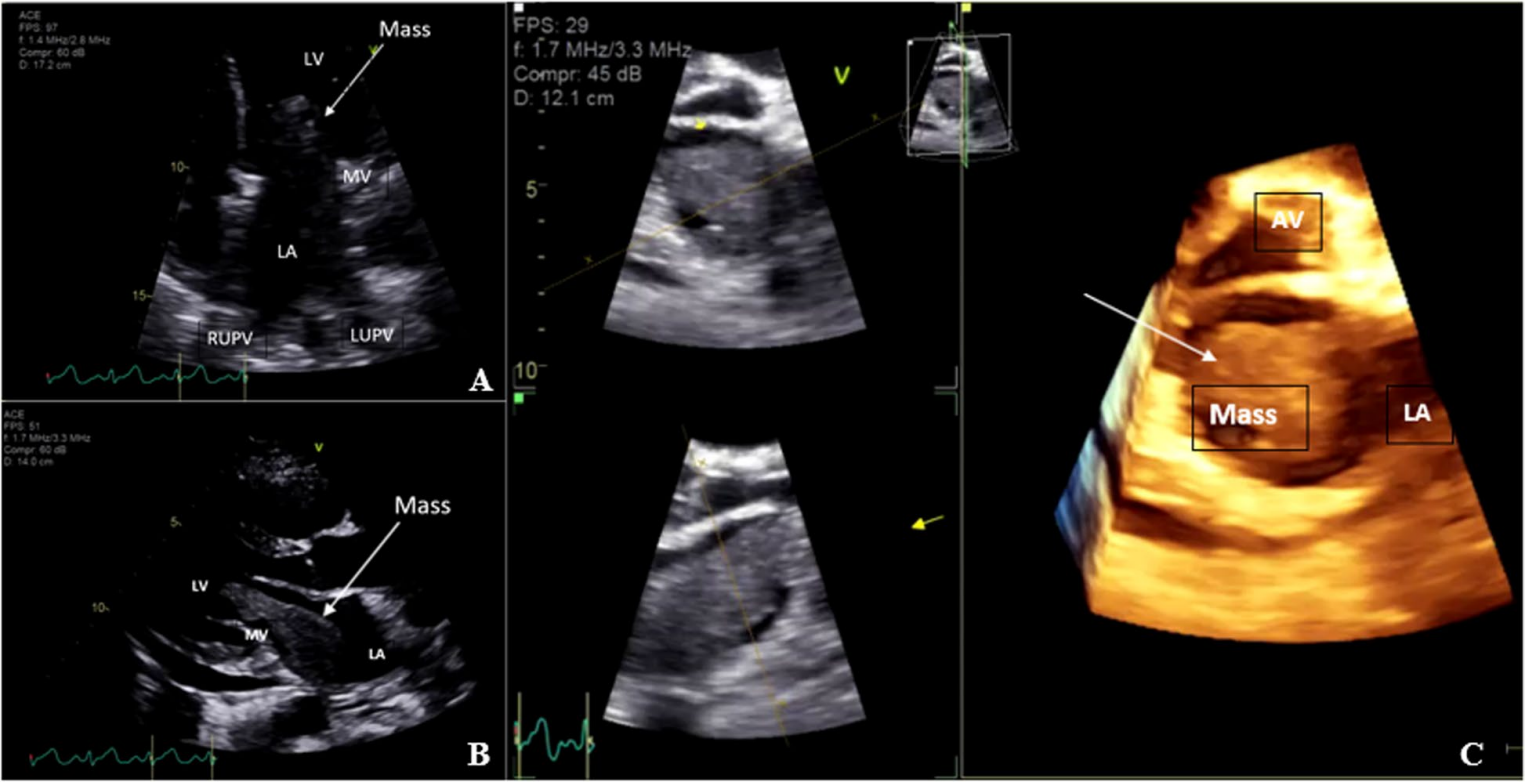
Transthoracic echocardiographic images of the left atrial mass. **A** 2D-echocardiography: Four chamber view demonstrates a large, echogenic mass attached to the lateral left atrial wall, prolapsing across the mitral valve causing left ventricular inflow obstruction. LA = left atrium, LV = left ventricle, MV = mitral valve, RUPV = right upper pulmonary vein, LUPV = left upper pulmonary vein. **B** 2D-echocardiography: Parasternal long axis view demonstrating obstruction of LV inflow with the mass prolapsing from the left atrium, across the mitral valve and into the left ventricle. **C** 3D-echocardiography demonstrating a large, well-circumscribed, echogenic mass (white arrow) filling the left atrium (LA). The aortic valve (AV) is oriented at the 12 o’clock position

**Fig. 3:**
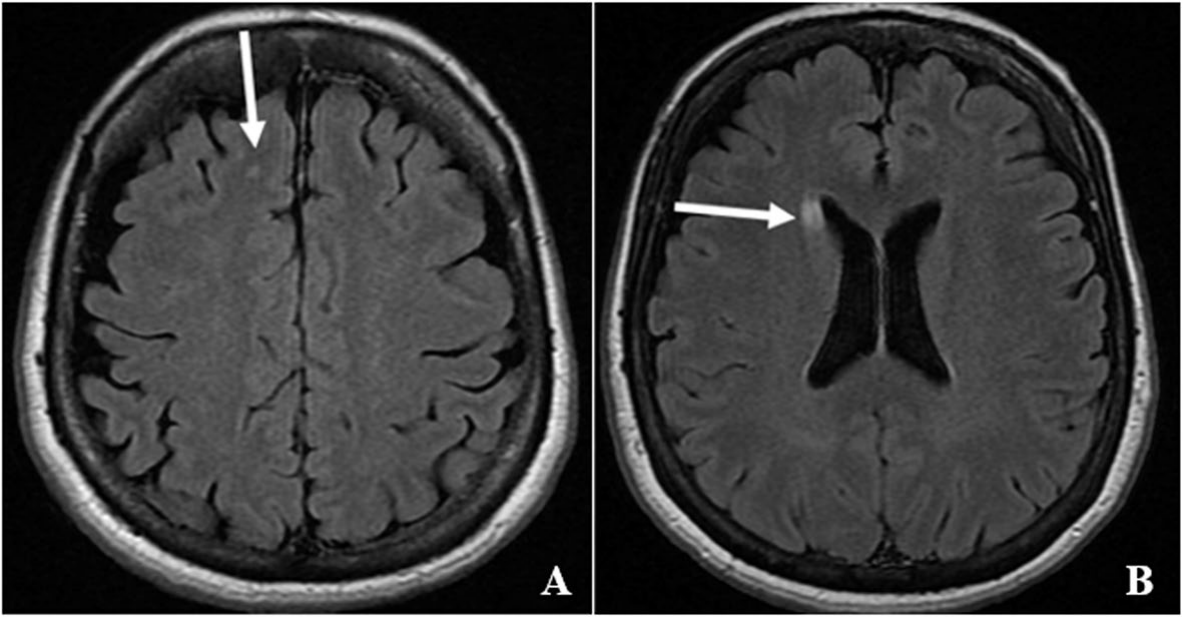
Transverse T2-FLAIR magnetic resonance images of the brain showing probable embolic lesions (white arrows) of variable age in the **A** right frontal lobe, and **B** right caudate head

Following urgent admission, investigations showed an increased CRP of 207 mg/L and an ESR of 104 mm/hr. There was a microcytic hypochromic anaemia with a haemoglobin of 80 g/L (normal 130-180 g/L) and a low serum albumin of 24 g/L (normal 36–40 g/L). Repeat ANA, ENA, RF, anti-CCP antibody, anti-phospholipid antibodies, anti-neutrophil cytoplasmic antibody and double-stranded dsDNA were negative. Urine and blood cultures were negative, as were Covid-19 polymerase chain reaction on peripheral blood, anti-Streptolysin O titre, bartonella, Q-fever, mycoplasma and brucella serology. A haemolysis screen was negative. Hepatitis B core antibody and surface antibody were positive, but Hepatitis C and HIV antibodies were not detected. Serum procalcitonin was not elevated. Electrocardiography showed a sinus tachycardia. Plain radiographs of the hands, wrists and knees demonstrated no erosions. Arthrocentesis of the left knee obtained fluid that was non-inflammatory, with a white cell count of 181 × 10^6/L (normal < 200 × 10^6/L), with no crystals, tumour cells or organisms seen. Bibasal pleural effusions were noted on a chest radiograph. A computerised tomography (CT) scan of the abdomen and pelvis was normal. Due to concern about infective endocarditis, urgent transthoracic echocardiography was performed and demonstrated a large villous mass with frond-like projections attached to the posterior left atrial (LA) wall, extending across the mitral valve (MV) with prolapse into the left ventricle (LV) in diastole. The LA was dilated and there was a small pericardial effusion (Fig. 2). Echocardiographic appearances were suggestive of an atrial myxoma with significant LV inflow obstruction. There were no other valvular lesions. Due to Covid-19, a cardiac magnetic resonance imaging (MRI) scan was unable to be performed. A chest CT demonstrated extension of the mass into the pulmonary veins. Coronary angiography was normal. Due to concern about possible emboli, a brain MRI was performed and showed multiple small right sided infarcts of differing ages (Fig. 3).

Because of the mitral inflow obstruction and the potential for further cerebral embolisation, emergency resection of the LA tumour and reconstruction of the LA and MV repair was performed via median sternotomy. At operation, a lobulated mass was found to arise from the posterior LA wall with a broad base between the left and right pulmonary vein origins with prolapse into the MV orifice. A separate drop lesion was found on the posterior MV leaflet. The tumour had encased the entire lumen of the left upper pulmonary vein and extended into the segmental branches - necessitating a left upper lobectomy. The left lower pulmonary vein required reconnection to the LA using a 14 mm expanded polytetrafluoroethylene interposition graft due to involvement of the proximal left lower pulmonary vein. The posterior wall of the LA was reconstructed, and the posterior MV leaflet repaired with autologous pericardial patches and placement of a mitral annuloplasty ring. The postoperative course was complicated by acute renal impairment and pulmonary oedema partly related to transfusion-related lung injury and a systemic inflammatory response. The patient was weaned off cardiopulmonary bypass and made an uncomplicated recovery.

**Fig. 4:**
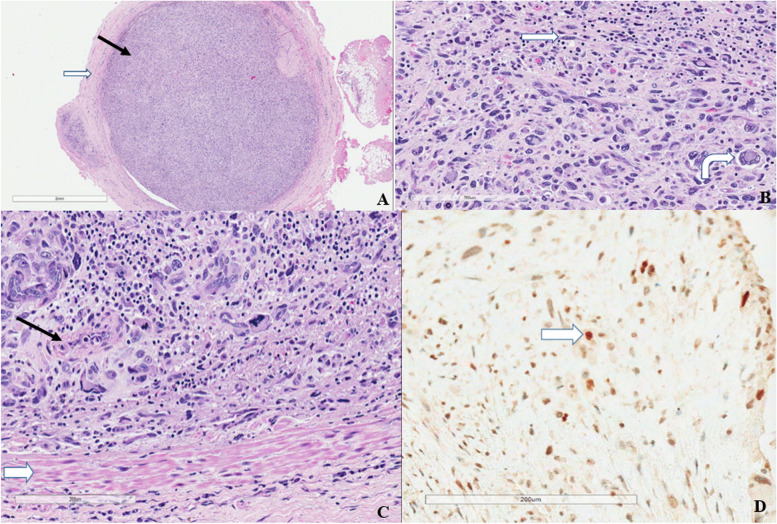
**A** Transverse section of pulmonary vessel with near complete occlusion of lumen (black arrow) by tumour. The tumour is confined within the tunica media (white arrow) with no invasion through the vessel wall. Haematoxylin and eosin stain, x1 magnification. **B** Sheets of pleomorphic tumour cells with admixed spindled cells (straight white arrow) and large multinucleate cells (bent white arrow). Haematoxylin and eosin stain, x10 magnification. **C** Tumour within pulmonary vessel (black arrow) with surrounding tunica media (white arrow). Haematoxylin and eosin stain, x10 magnification. **D** Tumour cells with positive staining for Murine Double Minute Clone 2, ranging from a weak blush to focally strong nuclear staining (white arrow). Haemotoxylin stain with bluing reagent, x20 magnification

Histopathology of the cardiac mass was consistent with a Grade 3 LA intimal (spindle cell) sarcoma (Fig. 4A-C) with positive staining for Murine Double Minute Clone 2 (MDM2) (Fig. 4D), an oncogene amplified in intimal sarcomas [4]. No myocardial or left upper lobe lymph node invasion was seen. One-month post-surgery, fluorodeoxyglucose - positron emission tomography (PET) revealed no residual glucose-avid tumour or extra-thoracic metastatic disease. The patient was commenced on combination chemotherapy with doxorubicin and isofosfamide. Complete resolution of joint pain and swelling and fingernail clubbing occurred within 2 weeks of tumour resection and has not returned. A recent surveillance brain MRI demonstrated a 3 mm T2-hyperintensity of the right caudate suggestive of previous infarct, but no definite metastases. There has been no deterioration in cardiac function. At time of writing, she is awaiting a surveillance PET scan.

## Discussion

This patient’s cardiac sarcoma presented with paraneoplastic arthropathy and finger clubbing. Symptom onset can precede malignancy detection by some years, although the diagnosis of malignancy can occur concurrently with joint manifestations - as seen in our case [5]. However, despite marked joint swelling, the knee synovial fluid in our case was non-inflammatory. While the exact pathogenesis is unknown, cross-reactivity of cancer cells and synovial antigens, toxic synovial effects from tumour cells, or concurrent independent effects of a shared casual factor may occur [6, 7]. Clinical features suggesting a paraneoplastic phenomenon are acute onset, asymmetrical joint involvement, a preference for lower limb joints, lack of auto-antibodies, lack of radiographic erosions, and accompanying systemic features, such as unintentional weight loss [8]. Age of onset over 50 years, lack of response to conventional treatment, including corticosteroids, should alert the clinician to consider a paraneoplastic arthritis [9]. In our patient, the lack of response to low-dose prednisolone expedited a face-to-face review despite strict Covid-19 pandemic restrictions. Symptom resolution often occurs with treatment of the underlying malignancy. Malignancies that arise from the colon, breast, ovaries, lung, stomach and haematolymphoid system are particularly associated with paraneoplastic arthropathy [9, 10].

A prominent feature of our patient’s presentation was digital clubbing, which resolved with tumour resection. Clubbing may be related to release of vasodilators in response to hypoxaemia. Platelet-rich micro-thrombi from the mass fragment in the peripheral circulation, release of platelet-derived growth factor and neural mechanisms may be implicated [11]. These factors result in oedema and increased vascularity of the connective tissue of the terminal phalanx, leading to the classical signs of acropathy [11, 12].

There is only one published case report of cardiac sarcoma presenting as paraneoplastic arthritis [3]. The histology was also of an intimal sarcoma with positive MDM2 staining. Primary cardiac tumours are extremely rare, and only 15% are malignant, such as sarcomas [1, 13]. The rate of primary cancers metastasizing to the heart is up to thirty times more common than primary cardiac tumours [14]. Benign cardiac tumours, such as atrial myxomas are more prevalent and are an important differential diagnosis, as they can also present with constitutional symptoms. In our case, echocardiographic appearances of the mass were classic for an atrial myxoma, highlighting the role of cardiac MRI for tissue characterisation and differentiation between the two entities. However, inability to access a cardiac MRI due to Covid-19 restrictions and the high risk of LV inflow obstruction and thromboembolic potential of the mass necessitated urgent surgery without an MRI. However, in less urgent situations, tissue characterisation with cardiac MRI is important to plan surgery.

Due to their rarity, there is conflicting literature on the frequency of intimal cardiac sarcomas, but most case series report them as infrequent [2, 4]. Complete surgical resection is the main treatment. There is scarce published evidence regarding adjuvant chemotherapy or radiotherapy, although 1-year survival rates are higher with a multimodal approach compared to surgery alone [15]. Increased survival with combination therapy has not been shown at 3 years post-diagnosis, and most patients die within a few years [16]. Given the high risk of recurrence and local spread, following review of data from adjuvant sarcoma studies and after multi-disciplinary discussions, our patient was given anthracycline- and ifosfamide-containing chemotherapy.

## Conclusion

This patient’s atypical presentation of corticosteroid-resistant arthropathy with clubbing led to detection and urgent removal of a rare cardiac sarcoma. With increasing telehealth consultations due to the Covid-19 pandemic, this case also highlights the importance of a detailed in-person clinical assessment.

## Data Availability

Not applicable.

## Abbreviations

CRP: C-reactive protein
ESR: Erythrocyte sedimentation rate
RF: Rheumatoid factor
anti-CCP: Anti-cyclic citrullinated peptide
ANA: Antinuclear antibodies
ENA: Extractable nuclear antigens
CT: Computerised tomography
LA: Left atrial
MV: Mitral valve
LV: Left ventricle
MRI: Magnetic resonance imaging
MDM2: Murine Double Minute Clone 2
PET: Positron emission tomography
RUPV: Right upper pulmonary vein
LUPV: Left upper pulmonary vein
AV: Aortic valve
